# DESIGN AND DEVELOPMENT OF VIRTUAL REALITY APPLICATION: AGE-FRIENDLY HEALTH SYSTEM SCENARIO

**DOI:** 10.1093/geroni/igad104.2612

**Published:** 2023-12-21

**Authors:** Samrah Mitha, Marie Dezine, Sweta Tewary, Vincent Guida, Hadi Masri, Naushira Pandya, Elizabeth Oviawe

**Affiliations:** Nova Southeastern University, Davie, Florida, United States; Nova Southeastern University, Davie, Florida, United States; Nova Southeastern University, Davie, Florida, United States; Nova Southeastern University, Davie, Florida, United States; Nova Southeastern University, Davie, Florida, United States; Nova Southeastern University, Kiran Patel College of Osteopathic Medicine, Davie, Florida, United States; Nova Southeastern University, Davie, Florida, United States

## Abstract

With an increase in the aging population, evidence-based practice education can help strengthen knowledge, skills, and clinical practice for healthcare students caring for older adults. Age Friendly Health Systems (AFHS) is one of the frameworks founded on providing evidence-based and low-risk care centered on what matters most to older adults, their families, and caregivers. While didactic education is still the primary force in teaching medical students, innovative methods are being included in the medical curriculum to practice competency. Virtual Reality (VR) platforms are popular due to their ability to provide an immersive hands-on learning experience resembling an actual medical practice. The immersion abilities engage the students’ sensory organs promoting an innovative way of learning concepts that are difficult to teach in real life. The project’s objective was to develop and publish a case highlighting AFHS that can help educate medical students on the 4Ms (Mobility, Mentation, Medication Management, and What Matters). The case was completed in multiple phases 1) using a design document modified through multiple edits and reviews by our interprofessional team; 2) technical development including environment, objects, and interfaces of the application; and 3) publication. The VR case displays four scenarios where an elderly patient is admitted for a hip fracture. Students learned how to triage and treat patients from admission to discharge while demonstrating their knowledge of AFHS. The case is integrated in the geriatric curriculum for medical students, and the education is ongoing. Future studies will evaluate the learning outcomes of medical students.

